# Perinatal depression impairs placental angiogenesis and fetal growth in mice: protective effects of betaine supplementation

**DOI:** 10.3389/fpsyt.2026.1769596

**Published:** 2026-04-22

**Authors:** Shuqin Jia, Guangjun Zhu, Hui Yang, Jing Chen, Tao Zou

**Affiliations:** 1 Department of Clinical Medicine, Guizhou Medical University, Guiyang, China; 2 Department of Psychiatry, Guiyang First People’s Hospital, Guiyang, China; 3 Department of Psychiatry, The Affiliated Hospital of Guizhou Medical University, Guiyang, Guizhou, China

**Keywords:** angiogenesis, betaine, fetal growth restriction, hypoxia, perinatal depression, placenta, VEGF

## Abstract

**Introduction:**

Perinatal depression (PND) is the most common psychiatric disorder experienced during pregnancy and postpartum and is characterized by persistent low mood, difficulty adapting to gestational changes, helplessness, social withdrawal, and, in severe cases, suicidal or infanticidal thoughts. In this study, using a mouse model of PND, we examined the impact of PND on placental angioneurotrophic signaling, namely vascular endothelial growth factor (VEGF), VEGF receptor-1 (VEGFR1), and brain-derived neurotrophic factor (BDNF), and determined whether placental hypoxia precipitates impaired angiogenesis and fetal growth restrictions (FGR). Additionally, we evaluated the therapeutic potential of betaine in restoring placental function and fetal growth.

**Methods:**

To establish a PND model, pregnant C57BL/6 mice (n = 40) underwent 6 weeks of pre-mating chronic unpredictable mild stress (CUMS) combined with social isolation, continued until gestational day (GD) 14; non-stressed littermates served as controls (n = 10). Model dams were randomized to receive physiological saline vehicle (10 mL/kg/day, DD, n = 10), low-dose betaine (50 mg/kg/day, DD+LB, n = 10), high-dose betaine (200 mg/kg/day, DD+HB, n = 10), or escitalopram (10 mg/kg/day, DD+ESC, n = 10). Stress and treatments continued until gestational day (GD) 17. On GD 18, fetal and placental weights were recorded and placental efficiency calculated. Placental levels of VEGF, VEGFR1, BDNF, and hypoxia-inducible factor-1α (HIF-1α) were quantified using reverse transcription quantitative polymerase chain reaction and western blot. Microvascular density (MVD) and vascular architecture were assessed using CD34 immunohistochemistry and hematoxylin–eosin staining.

**Results:**

CUMS with social isolation evoked robust depression- and anxiety-like behaviors and attenuated maternal weight gain, thereby validating the PND model. PND resulted in significant fetal growth restriction, decreased placental efficiency, and marked reductions in placental BDNF and VEGF. These alterations coincided with elevated HIF-1α, indicating placental hypoxia. High-dose betaine reversed these deficits, restoring fetal weight and placental efficiency to control levels while upregulating placental BDNF and VEGF expression. Histologically, high-dose betaine increased MVD and improved vascular perfusion.

**Conclusions:**

These findings highlight betaine as a promising, low-risk intervention for preventing PND-associated fetal complications.

## 1 Introduction

Perinatal depression (PND) is the most common psychiatric disorder experienced during pregnancy and postpartum (1) and is characterized by persistent low mood, failure to adapt to gestational changes, helplessness, social withdrawal, and in severe cases, suicidal or infanticidal thoughts (2). Maternal depressive symptoms can be transmitted to the next generation biologically and psychologically (3–8), and the rising incidence highlights the urgent need for research (9–11). Current guidelines recommend non-pharmacological interventions, which are often ineffective in moderate-to-severe cases (9–11). Untreated PND endangers mother and child, causing preterm birth, low birthweight, impaired bonding, and long-term cognitive, emotional, and behavioral dysregulation that may reach the third generation (12–17). Pharmacotherapy is limited by teratogenic risks, leading many women to refuse treatment (18); hence, developing safe and effective strategies remains an unmet need. Elucidating the molecular mechanisms of PND-related affective dysregulation and its transgenerational impact is essential for rational drug design.

Intrauterine growth restriction is closely linked to placental insufficiency, characterized by reduced syncytiotrophoblast surface, thickened trophoblast–capillary diffusion barrier, and decreased vascular density (19–21), with aberrant placental angiogenesis being the primary driver (22). As the placenta maintains a high-flow system for fetal nutrient and oxygen delivery (23, 24), defective vascularization reduces placental efficiency, lowers fetal weight, decreases litter size in mice, and impairs growth (25–27).

Vascular endothelial growth factor (VEGF) and brain-derived neurotrophic factor (BDNF) are key regulators of placental vascular development. The VEGF family (PlGF, VEGF-A, VEGF-B, VEGF-C, and VEGF-D) and fibroblast growth factor (FGF)/angiopoietin systems coordinate angiogenesis via VEGFR-1/2 on placental endothelium (28–30). Although both VEGFR1 and VEGFR2 are expressed in placental endothelium, we focused on VEGFR1 (Flt-1) because it serves as a decoy receptor that modulates VEGF availability and has been implicated in placental pathologies such as preeclampsia and intrauterine growth restriction. VEGFR1 lacks a functional kinase domain and primarily acts as a negative regulator of angiogenesis by sequestering VEGF, whereas VEGFR2 mediates the pro-angiogenic signaling. Dysregulation of the VEGF-VEGFR1 axis has been specifically associated with placental hypoxia and vascular dysfunction. Beyond angiogenesis, maternal BDNF can cross the placenta and modulate fetal development (31). BDNF exerts its biological effects primarily through binding to the tropomyosin receptor kinase B (TrkB). BDNF-TrkB signaling promotes endothelial cell proliferation, migration, and survival in the placenta, and reduced placental BDNF has been associated with impaired angiogenesis in gestational diabetes mellitus. However, the role of placental BDNF/TrkB signaling in perinatal depression remains unexplored.

Dysregulation of angioneurotrophic factors has been implicated in depression, and VEGF levels increase during acute episodes (32, 33). However, one study showed that although depressive symptoms improved following treatment, patients’ serum VEGF levels showed no significant change compared with pre-treatment values (34). In addition, paradoxically, VEGF levels are decreased in completed suicides (35). Similarly, BDNF was found to be reduced in the postmortem hippocampus and prefrontal cortex of patients with major depressive disorder (36), and chronic stress is known to lower hippocampal BDNF and trigger depressive-like behavior in animals (36).

Botanical alkaloids such as aconitine, berberine, scopolamine, and total alkaloids from *Ziziphus jujuba* have demonstrated antidepressant effects in rodents (37–39). By enhancing S-adenosyl-L-methionine (SAMe) synthesis, betaine (a natural alkaloid) lowers homocysteine (Hcy), a depression biomarker correlated with symptom severity (40, 41), and it has been shown to alleviate mild-to-moderate treatment-resistant depression (42). Notably, betaine is a major bioactive constituent isolated from *Lycium barbarum* root/bark, which has been traditionally used for its antidepressant effects in Chinese medicine (43). Betaine also counteracts oxidative stress by scavenging radicals, upregulating superoxide dismutase (SOD), downregulating malondialdehyde (MDA), and modulating Bcl-2/Bax to promote endothelial proliferation, migration, and angiogenesis while suppressing pathological neovascularization via reactive oxygen species (ROS)–mediated VEGF signaling (44, 45). However, the role of betaine in placental angiogenesis during PND remains unexplored. Maternal depression–driven Hcy elevation may injure placental endothelium and promote thrombosis, whereas betaine-mediated Hcy reduction could preserve placental vascular integrity. Therefore, we aimed to examine whether betaine ameliorates PND-associated fetal growth restriction (FGR) via modulation of placental angioneurotrophic factors in a chronic unpredictable mild stress (CUMS) plus single-housing mouse model of PND. Escitalopram, a selective serotonin reuptake inhibitor (SSRI), was selected as a positive control to compare betaine’s efficacy against standard pharmacotherapy for perinatal depression. As a first-line antidepressant for PND (46), escitalopram has a relatively favorable safety profile among SSRIs during pregnancy, and it has established transplacental transfer characteristics (18). This comparison enables an evaluation of betaine as a potentially safer alternative with fewer teratogenic concerns.

## 2 Materials and methods

### 2.1 Animals and grouping

All animal procedures were reported in accordance with the ARRIVE (Animal Research: Reporting of *In Vivo* Experiments) 2.0 guidelines to ensure transparency, reproducibility, and ethical completeness of the study. Fifty female C57BL/6 mice (18 ± 2 g) were supplied by the Laboratory Animal Center of Guizhou Medical University and maintained on a 12 h light–dark schedule (lights on at 08:00, consistent with standard housing conditions to minimize additional stressors), at 22 ± 2 °C and 50 ± 12% relative humidity, and with unrestricted access to standard chow and water. All procedures were approved by the Institutional Animal Ethics Committee (approval no. 2201333) and conducted in strict accordance with the relevant guidelines. Following 7 days of acclimation, 10 mice remained group-housed (5 per cage) and served as the control cohort, while the remaining 40 were single-caged and exposed to 6 weeks of CUMS plus social isolation to induce a depressive-like phenotype. Behavioral validation (pre- and post-CUMS) was ascertained using the sucrose preference, forced-swim, and open-field assays. Following validation, the stressed mice were redistributed into four equal arms (n = 10 per group): DD (depression-model dams receiving physiological saline vehicle, 10 mL/kg/day), DD+HB (200 mg/kg betaine), DD+LB (50 mg/kg betaine), and DD+ESC (10 mg/kg escitalopram). These doses were selected based on preliminary experiments in our laboratory to establish a dose–response relationship for betaine in this model. The low dose (50 mg/kg) represents a suboptimal dose to test dose-dependent effects, while the high dose (200 mg/kg) was chosen as the maximal effective dose without observed toxicity in pilot studies. The dose of 10 mg/kg/day was selected based on previously published preclinical studies demonstrating antidepressant efficacy in rodent models of depression (47). Escitalopram, a selective serotonin reuptake inhibitor (SSRI) and first-line antidepressant for PND (46), was included as a positive control to compare betaine’s efficacy against standard pharmacotherapy. While SSRIs cross the placenta, escitalopram is considered to have a relatively favorable safety profile among antidepressants during pregnancy (18), making it an appropriate comparator for evaluating betaine as a potentially safer alternative. Subsequently, females were mated with non-stressed, proven fertile C57BL/6 males (2:1 ratio) that were group-housed under standard conditions without exposure to CUMS or social isolation. Female reproductive cycles were monitored by vaginal cytology for 2 weeks prior to mating to ensure regular estrous cycles; only females showing consistent 4–5 day cycles were included in the study. Only females with confirmed vaginal plugs (indicating successful mating) were included in the study; non-pregnant females were excluded. The presence of a vaginal plug was defined as gestational day 0 (GD 0, the morning of vaginal plug detection). For clarity in timeline tracking, subsequent days are referred to as GD 1, GD 2, etc. CUMS exposure continued until GD 14. Pharmacological treatment (betaine, escitalopram, or physiological saline vehicle) was administered daily by oral gavage, starting from GD 0 and continuing through GD 18. On GD 18, placentas and fetuses were harvested, weighed, and placental efficiency (fetal weight/placental weight) was computed.

CUMS exposure was terminated at GD 14 to minimize acute stress-induced abortion risk during the vulnerable early-mid gestation period while maintaining the depressive phenotype (48). Pharmacological treatment continued until GD 18 to ensure therapeutic coverage during the critical window of rapid fetal growth and peak placental angiogenesis (49). The experiment was concluded at GD 18 because (i) this stage represents maximal placental vascular development suitable for assessing angiogenic mechanisms (50); (ii) GD 18 in mice corresponds to the third trimester in humans, the period most relevant for FGR (50).; (iii) tissue collection at this stage avoids confounding factors from parturition stress and postpartum hormonal changes (51); and (iv) in most cases, the freshly delivered placenta is eaten by the dams, which affects the acquisition of placental samples (52).

### 2.2 CUMS paradigm

The CUMS protocol was adapted from established procedures originally described by Willner, et al. (53) and subsequently optimized for C57BL/6 mice (54). A 6-week CUMS paradigm was implemented using nine randomly sequenced stressors per day: (i) immersion in 42–45 °C water for 5 min; (ii) platform oscillation until postural collapse (5 min); (iii) 1-min tail clip; (iv–v) 24-h water or food withdrawal; (vi) 2-h restraint in a 50-mL conical tube; (vii) 85-dB white noise for 30 min; (viii) inverted light–dark cycle for 12 h; and (ix) a 45°cage tilt for 24 h. The stressors were selected to be unpredictable and mild-to-moderate in intensity, based on published literature demonstrating their efficacy in inducing depressive-like phenotypes without causing severe physical harm. Specifically, water temperature (42–45 °C) was chosen based on protocols showing that this range effectively induces thermal stress without tissue damage, and 24-h food/water deprivation was included as an intermittent, non-continuous stressor, as per established CUMS paradigms.

The CUMS plus isolation model used in this study has been extensively validated in previous publications (53, 54), showing robust depressive-like behaviors consistent with human PND. While we focused on behavioral endpoints (anhedonia, behavioral despair, anxiety-like behaviors) and placental outcomes in this study, we acknowledge that circulating stress hormones were not measured. Previous studies using similar CUMS protocols in mice have demonstrated elevated serum corticosterone and adrenocorticotropic hormone (ACTH) (55), confirming stress induction. As such, future studies should include plasma corticosterone measurements to further validate the stress response in this model.

### 2.3 Behavioral testing

Behavioral assessments were conducted as previously described (54, 56, 57). Depression- and anxiety-like behaviors were evaluated before (baseline) and after CUMS using the sucrose preference test to evaluate anhedonia, the forced swim test to evaluate behavioral despair, and the open-field test to evaluate locomotor activity and anxiety. In the sucrose preference test, mice were habituated to two bottles of 1% sucrose solution for 48 h, followed by 24 h of water deprivation. Mice were then presented with two bottles containing 1% sucrose solution and regular water, respectively, for 24 h. Sucrose preference was calculated as [sucrose intake/(sucrose intake + water intake)] × 100%. In the forced swim test, mice were placed individually in a transparent cylinder (diameter 12 cm, height 25 cm) filled with water (23–25 °C) to a depth of 15 cm for 6 min. The duration of immobility (defined as floating without struggling) and active struggling time were recorded during the last 4 min by an observer blinded to group allocation. In the open-field test, mice were placed in the center of a square arena (50 × 50 × 40 cm) divided into central and peripheral zones. Locomotor activity (total distance traveled) and anxiety-like behavior (time spent and distance traveled in the central zone) were recorded over a 5-min period using an automated tracking system.

### 2.4 Tissue collection and processing

At day 18 of gestation, dams were anesthetized with isoflurane (4% for induction, 2% for maintenance, delivered via a precision vaporizer in a 0.8 L/min oxygen flow rate) and subsequently euthanized by cervical dislocation. All procedures were performed in accordance with institutional guidelines to minimize animal distress. Fetuses and placentas were rapidly removed and immediately placed on ice (0–4 °C) for temporary preservation prior to dissection and subsequent processing (within 30 min). For each litter, one placenta was immersion-fixed in neutral-buffered formalin (10%) prior to subsequent histological and immunohistochemical analysis. For histological examination, fixed tissues were dehydrated, embedded in paraffin, sectioned at 3 μm, and stained with hematoxylin and eosin (H&E) using standard protocols. H&E-stained sections were evaluated using a semi-quantitative scoring system by two independent observers blinded to group allocation. Vascular organization was scored as follows: 0 = regular arrangement, 1 = mild disorganization, 2 = moderate disorganization, 3 = severe disorganization. Blood cell content was scored as: 0 = abundant, 1 = moderately reduced, 2 = markedly reduced, 3 = absent. Total histopathological scores were compared among groups. All remaining tissues were snap-frozen in liquid nitrogen and stored at -80 °C prior to molecular analysis. Fetal and placental weights were recorded, and the efficiency index was calculated by dividing fetal mass by placental mass.

### 2.5 Western blotting

Protein extraction and immunoblotting were performed following standard protocols (58, 59). Total protein content was assayed using the bicinchoninic acid method (60). For immunoblotting, 50-mg snap-frozen placental fragments were lysed in ice-cold radioimmunoprecipitation (RIPA) buffer containing 1 mM phenymethylsulfonyl fluoride (PMSF) and a cocktail of protease and phosphatase inhibitors. Subsequently, 30-µg aliquots were separated on 10% sodium dodecyl sulfate polyacrylamide gel electrophoresis (SDS-PAGE) gels and electro-transferred onto polyvinylidene fluoride (PVDF) membranes. Following 1 h blocking in 5% non-fat milk, membranes were probed overnight at 4 °C with primary antibodies targeting VEGF (rabbit polyclonal, 1:2000, catalog Number: 19003-1-AP, Proteintech Group Inc.), VEGFR1 (rabbit monoclonal, 1:1000, catalog Number: R014248, Shanghai Yamay Biomedical Technology Co., Ltd.), BDNF (rabbit polyclonal, 1:500, catalog Number: 25699-1-AP, Proteintech Group Inc.), and β-actin (mouse monoclonal, 1:50000, catalog Number: 66009-1-lg, Proteintech Group Inc.). After 1 h of incubation with goat anti-mouse IgG (1:5000, catalog Number: RGAR001, Proteintech Group Inc.) at room temperature (20–25 °C), signals were developed with enhanced chemiluminescence (ECL) substrate (Thermo Fisher Scientific) and captured with a ChemiDoc XRS+ imaging system (Bio-Rad Laboratories, Hercules, CA), followed by densitometric analysis using ImageJ v1.54.

### 2.6 Quantitative real-time PCR

Quantitative real-time PCR (qPCR) was performed as previously described (61). Total RNA was extracted with TRIzol reagent (Thermo Fisher Scientific, Waltham, MA). RNA concentration and purity were assessed using a NanoDrop spectrophotometer (Thermo Fisher Scientific), and 1 μg of total RNA per sample was reverse-transcribed using a PrimeScript RT Reagent Kit (Takara Bio, Kusatsu, Japan). qPCR was performed on a CFX96 platform (Bio-Rad Laboratories, Hercules, CA) with SYBR Green Master Mix (Takara Bio). Primer pairs are listed in **Table 1**. Expression levels were calculated by the 2^−ΔΔCt^ method and normalized to *Gapdh*. Data are presented as fold change relative to the control group. qPCR was performed in triplicate for each sample using the following conditions: initial denaturation at 95 °C for 30 s, followed by 40 cycles of denaturation at 95 °C for 5 s, annealing at 60 °C for 30 s, and extension at 72 °C for 30 s. A melting curve analysis was performed to confirm primer specificity. HIF-1α mRNA levels were assessed by qPCR as an indicator of transcriptional activation under hypoxic conditions. While we acknowledge that HIF-1α is primarily regulated post-translationally, mRNA upregulation precedes protein stabilization and serves as an early marker of hypoxic response (62).

**Table 1:** Primer sequences used for quantitative real-time PCR.

Gene	Forward primer (5'-3')
GAPDH-F GAPDH-R VEGF-F VEGF-R VEGFR1-F VEGFR1-R BDNF-F BDNF-R HIF-1a-R HIF-1a-F	GCATCCTGGGCTACACTGAG GTCAAAGGTGGAGGAGTGGG GCACATAGAGAGATGAGCTTCC CTCCGATCTGAACAAGGCT TAAGCCTGGGGAACTCATTCT TAAGCCTGGGGAACTCATTCT TTACCTGGATGCCGCAAACAT TGACCCACTCGCTAATACTGTC ACTGTTAGGCTCAGGTGAACT ACCTTCATCGGAAACTCCAAAG

### 2.7 Immunohistochemistry

IHC was performed following standard protocols (63). Paraffin-embedded placental sections (3 μm) were dewaxed, rehydrated, and heat-treated for antigen retrieval (63). After quenching endogenous peroxidase with 3% H_2_O_2_, slices were probed overnight at 4 °C with rabbit anti-mouse CD34 monoclonal antibody (rabbit polyclonal, 1:500, catalog Number: 31120-1-AP, Proteintech Group Inc.) (64), incubated with HRP-labeled secondary antibody, and developed with DAB. Nuclei were counterstained with hematoxylin. Microvascular density (MVD) was estimated by counting CD34-positive vessels in six randomly chosen 200× fields per section, as described (65). CD34-positive vessels were defined as brown-stained endothelial structures with distinct lumens. Counting was performed independently by two observers blinded to group allocation using ImageJ software, and the average count per field was calculated for each placenta.

### 2.8 Statistical analysis

Data are presented as mean ± standard deviation. Normality was examined with the Shapiro–Wilk test, and multiple-group comparisons were performed using one-way analysis of variance followed by Tukey’s *post-hoc* procedure using SPSS version 26 (IBM, Armonk, NY). Figures were generated using GraphPad Prism 7 (GraphPad Software, San Diego, CA). Placental efficiency was calculated as the ratio of fetal to placental mass multiplied by 100. Statistical significance was defined as a two-tailed P-value < 0.05. Sample size was prospectively set via power analysis (α = 0.05, β = 0.8) using pilot fetal-weight values.

## 3 Results

### 3.1 Changes in body weight and behavior of mice exposed to CUMS combined with social isolation

#### 3.1.1 Body weight

Prior to modeling, baseline body mass did not differ between control and stressed mice (P > 0.05). Following 6 weeks of CUMS plus social isolation, stressed mice weighed markedly less than controls (P < 0.001). Additionally, during the modeling period, the model group showed a slower rate of weight gain (**Table 2**).

**Table 2:** Effects of chronic unpredictable mild stress on body weight in mice (g, X ± S).

Time	Control group (n = 10) Weight (g)	Model group (n = 40) Weight (g)
0 week	19.28 ± 0.167	19.18 ± 0.109
6 weeks	21.46 ± 0.257	19.41 ± 0.212***

***Compared with the control group at 6 weeks, P < 0.001.

#### 3.1.2 Sucrose preference test

**Figure 1:**
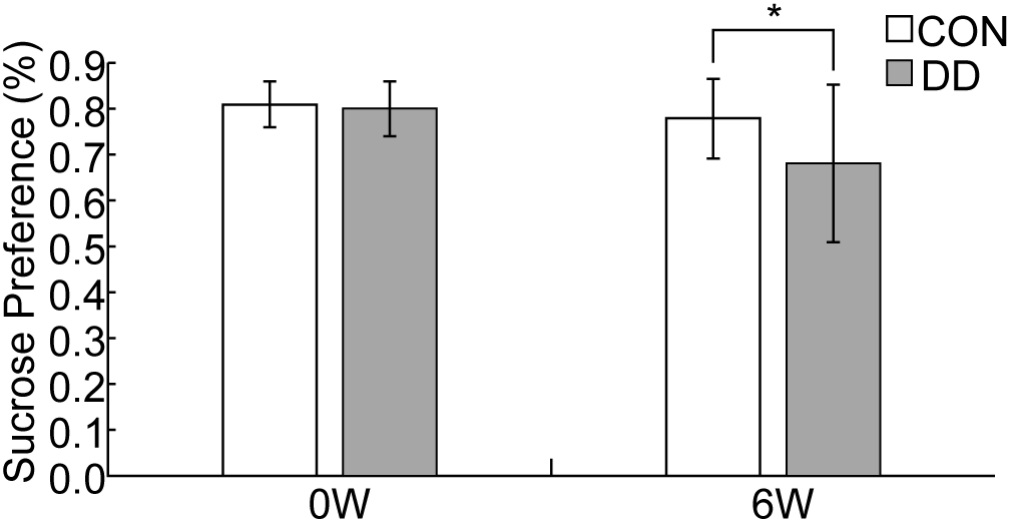
Changes in sucrose preference test in depressive mice. CON, control group; DD, model group. *Compared with the control group, P < 0.05.

Pre- modeling sucrose preference did not differ between control and model mice (P > 0.05). After 6 weeks of CUMS plus social isolation, the model group showed a significantly lower sucrose preference rate than that of the controls (P < 0.05, **Figure 1**).

#### 3.1.3 Forced swim test

**Figure 2:**
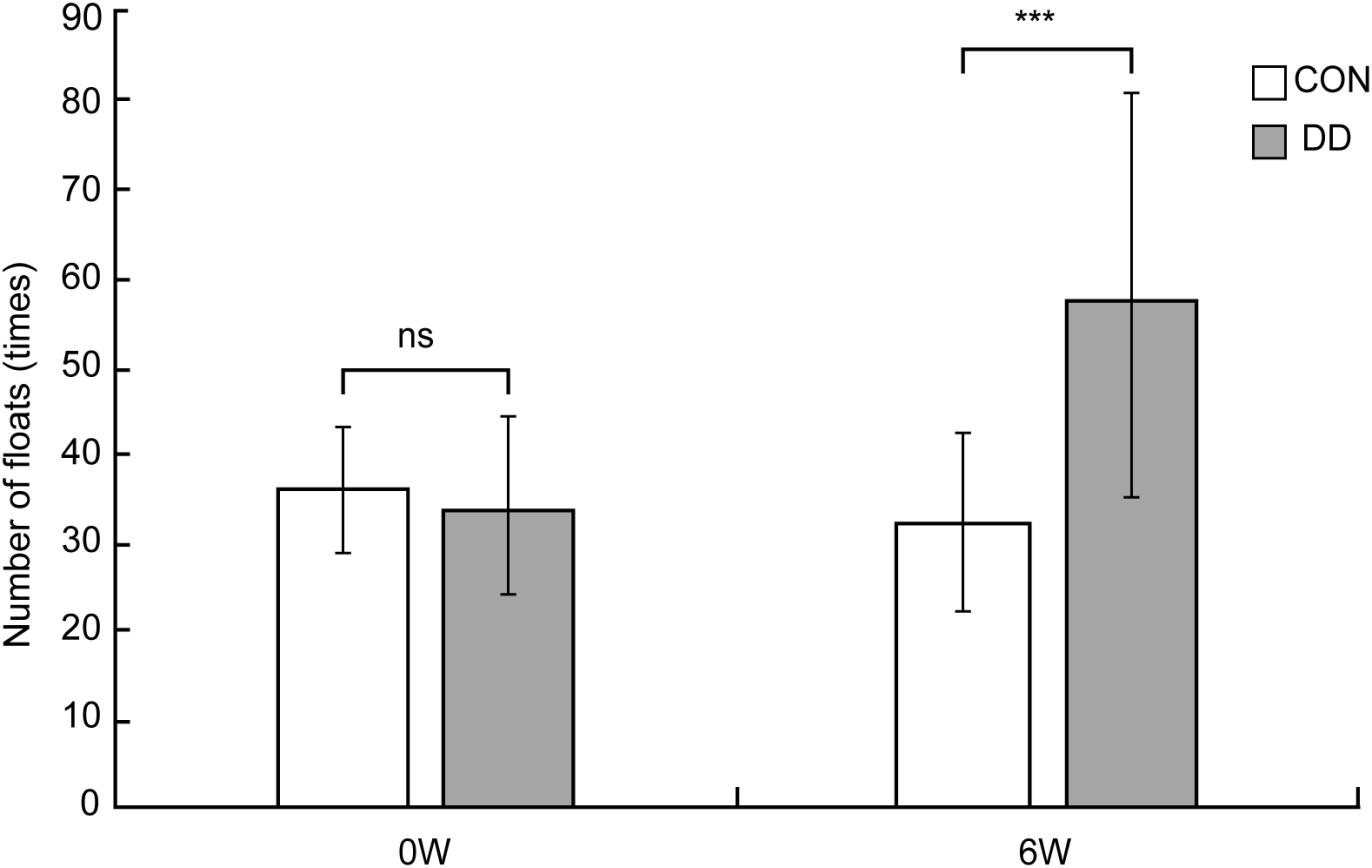
Number of forced swimming floats in depressive model mice. CON, control group; DD, model group. ***Compared with the control group, P < 0.001.

**Figure 3:**
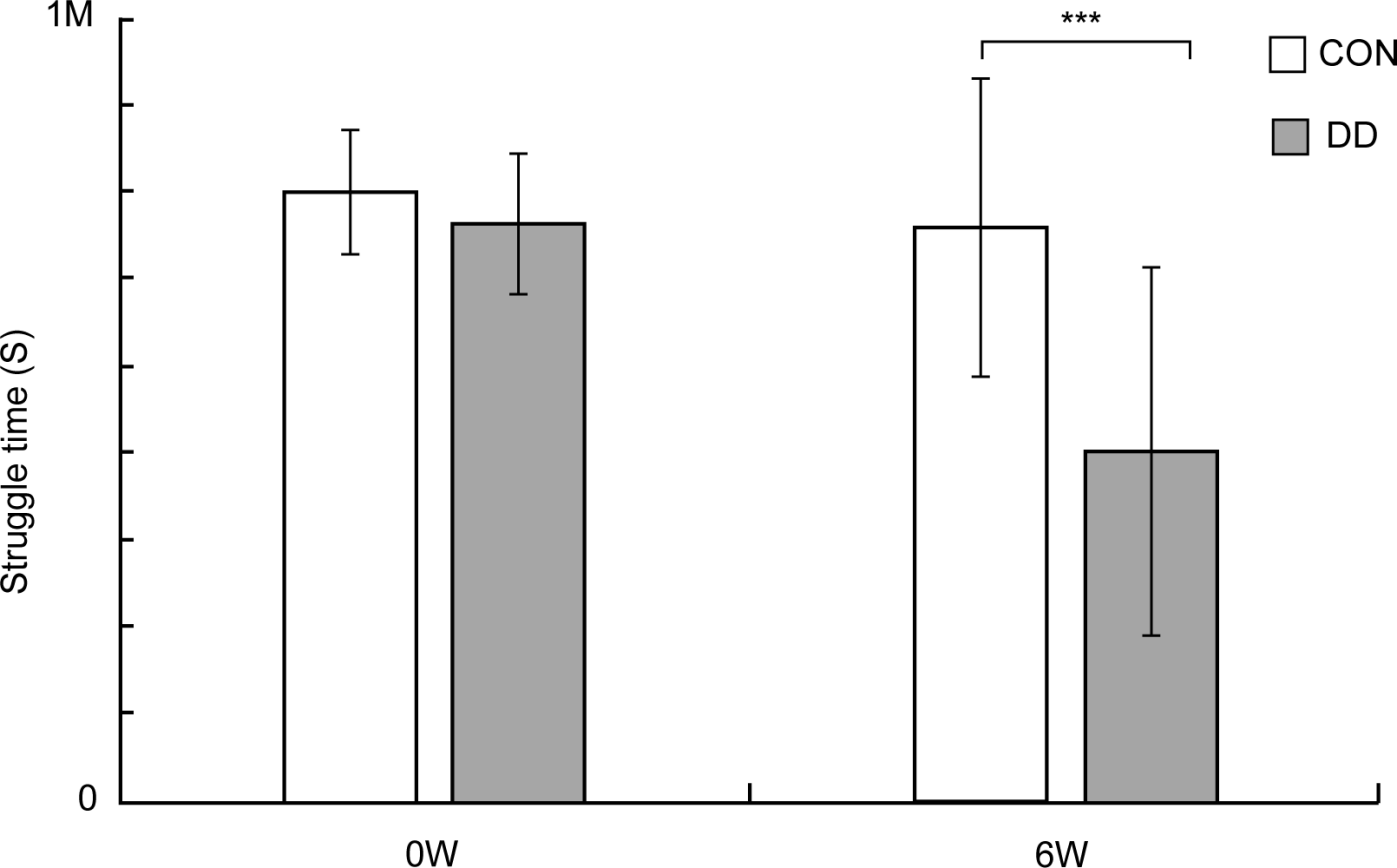
Forced swimming struggling time in depressive model mice. CON, control group; DD, model group. ***P < 0.001 vs. control group.

Prior to stress onset, no significant difference in immobility frequency and struggling time was observed between control and model mice (P > 0.05). Following 6 weeks of CUMS plus social isolation, the model group exhibited a significantly higher immobility frequency (P < 0.001, **Figure 2**) and a significantly shorter struggling time than that of controls (P < 0.001, **Figure 3**).

#### 3.1.4 Time spent in the central zone

**Figure 4:**
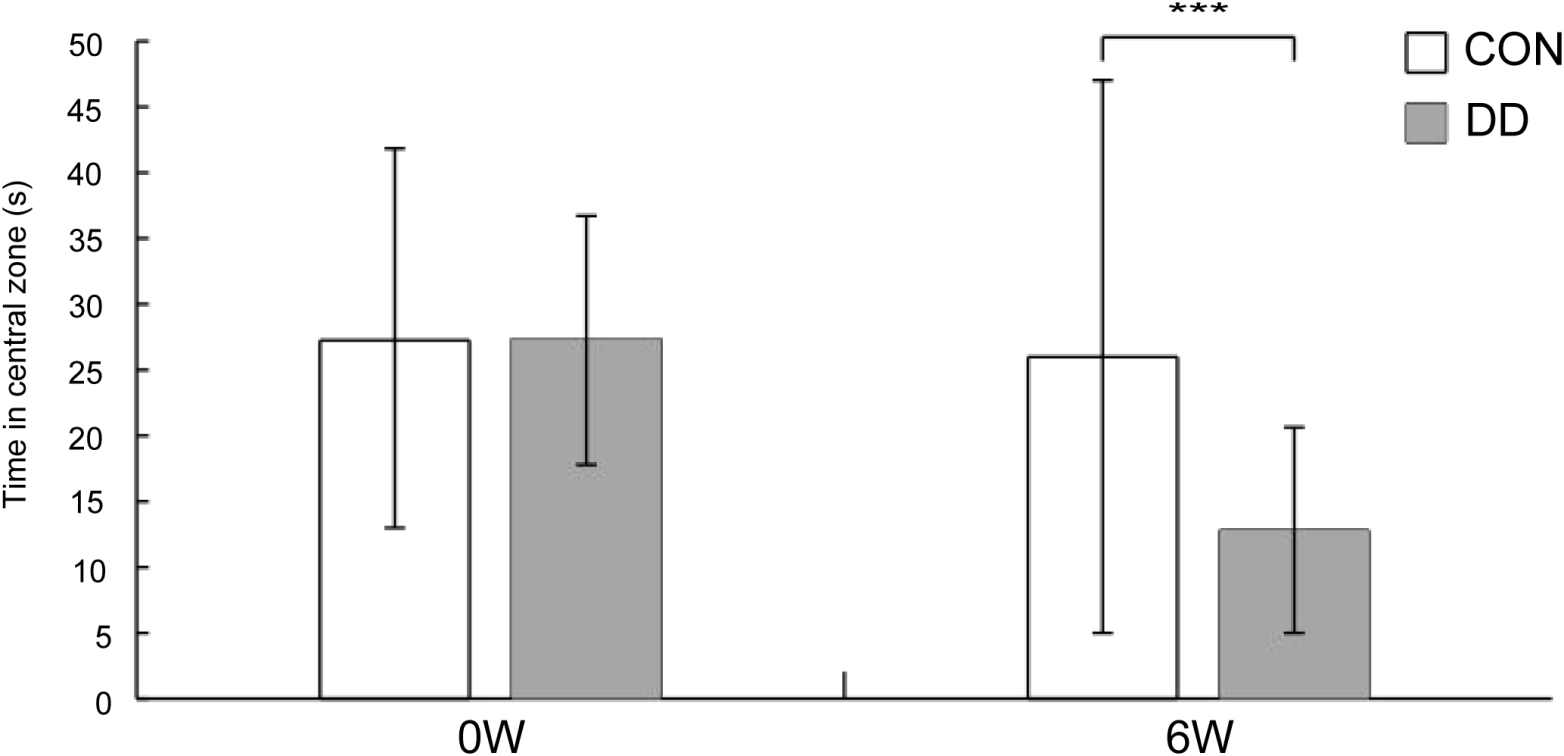
Time spent in the central area during the open-field test in depressive model mice. CON, control group; DD, model group. ***P < 0.001 vs. control group.

In the open-field test, before modeling, no significant difference was observed in the time spent in the central zone between the model and control groups. However, after 6 weeks of CUMS combined with social isolation, the model group exhibited a significantly reduced time spent in the central zone compared to the control group (P < 0.001) (**Figure 4**).

#### 3.1.5 Distance travelled in the central zone

**Figure 5:**
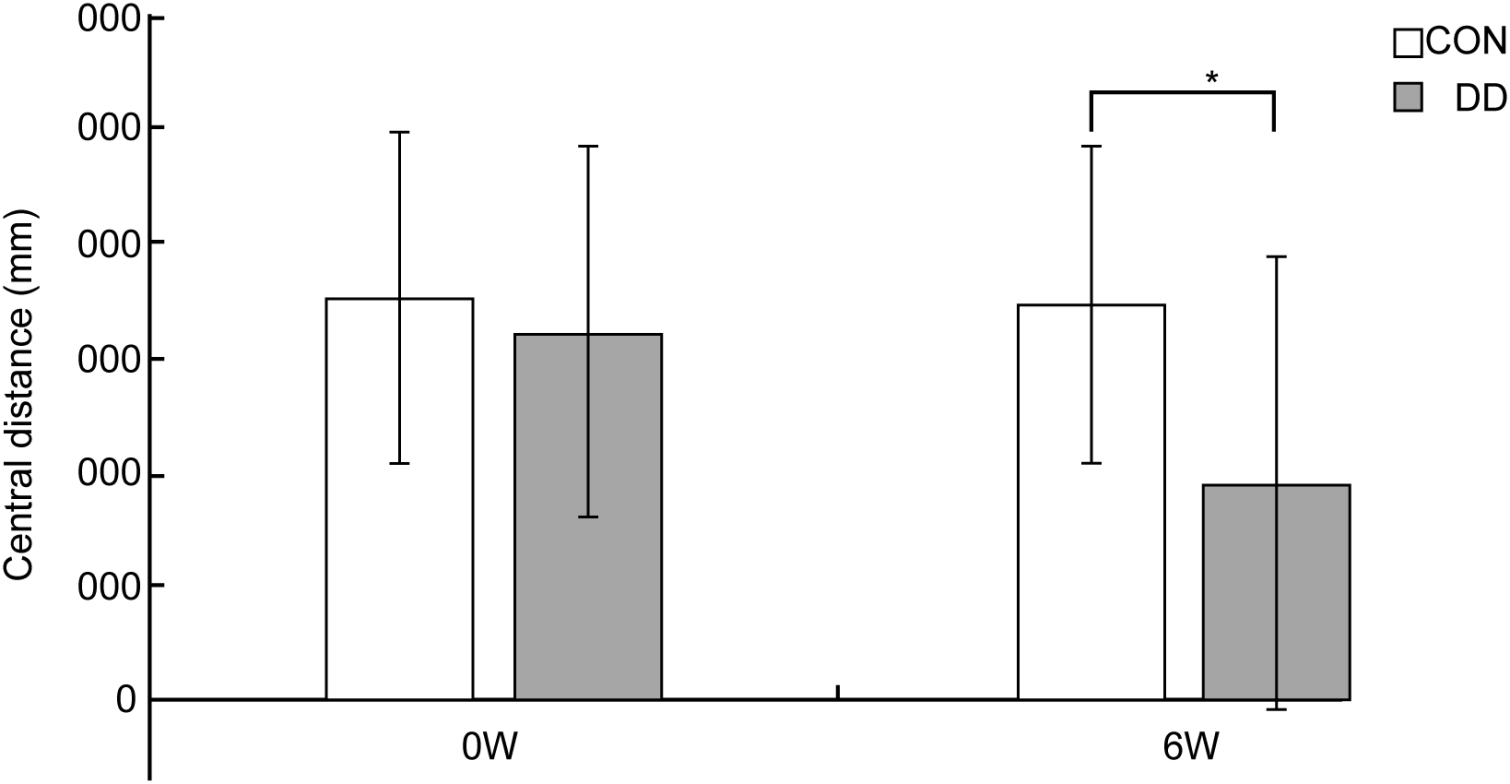
Distance covered in the central area by depressive model mice in the open-field test. CON, control group; DD, model group. *P < 0.05 vs. control group.

In the open-field test, pre- modeling central-zone distance did not differ between groups; however, after 6 weeks of CUMS plus social isolation, model mice covered significantly less distance in the central zone than controls (P < 0.05) (**Figure 5**).

### 3.2 Changes in fetal weight, placental weight, and placental efficiency

Stressed dams delivered significantly lighter fetuses than those of controls (P < 0.01). Compared with stressed dams, both the high- and low-dose betaine treatment groups showed a significant increase in fetal weight (P < 0.05). Compared with the DD model group, dams treated with escitalopram showed a numerical increase in fetal weight; however, this change did not reach statistical significance (P > 0.05). Placental weight tended to be lower in the model group than in controls; however, the difference was not statistically significant (P > 0.05). All three intervention groups gained some placental mass relative to stressed dams; however, none differed significantly (P > 0.05). Consequently, placental efficiency (fetal/placental ratio) remained depressed in the model cohort compared to controls (P < 0.05). In contrast, compared with the model group, the high-dose betaine, low-dose betaine, and escitalopram treatment groups showed no significant differences in placental efficiency (P > 0.05) (**Table 3**).

**Table 3:** Comparison of the weights of fetuses, placentas, and placental efficiency among five groups (X ± S).

Group	Fetal weight (mg)	Placental weight (mg)	Placental efficiency (%)
CON	1. 12 ± 0.178	0.096 ± 0.017	13.175 ± 4.363
DD	0.47 ± 0.150##	0.086 ± 0.011	5.083 ± 1.413#
DD+HB	1.06 ± 0.179*	0.089 ± 0.012	11.503 ± 2.201
DD+LB	1.00 ± 0.328*	0.092 ± 0.007	10.446 ± 3.844
DD+ESC	1.00 ± 0.260	0.089 ± 0.012	10.600 ± 3.131

*compared with the model group, P < 0.05.

^#^compared with the control group, P < 0.05.

^##^compared with the control group, P < 0.01.

### 3.3 PND mouse model: placental protein expression of VEGF, VEGFR1, and BDNF

#### 3.3.1 Placental VEGF protein expression

**Figure 6:**
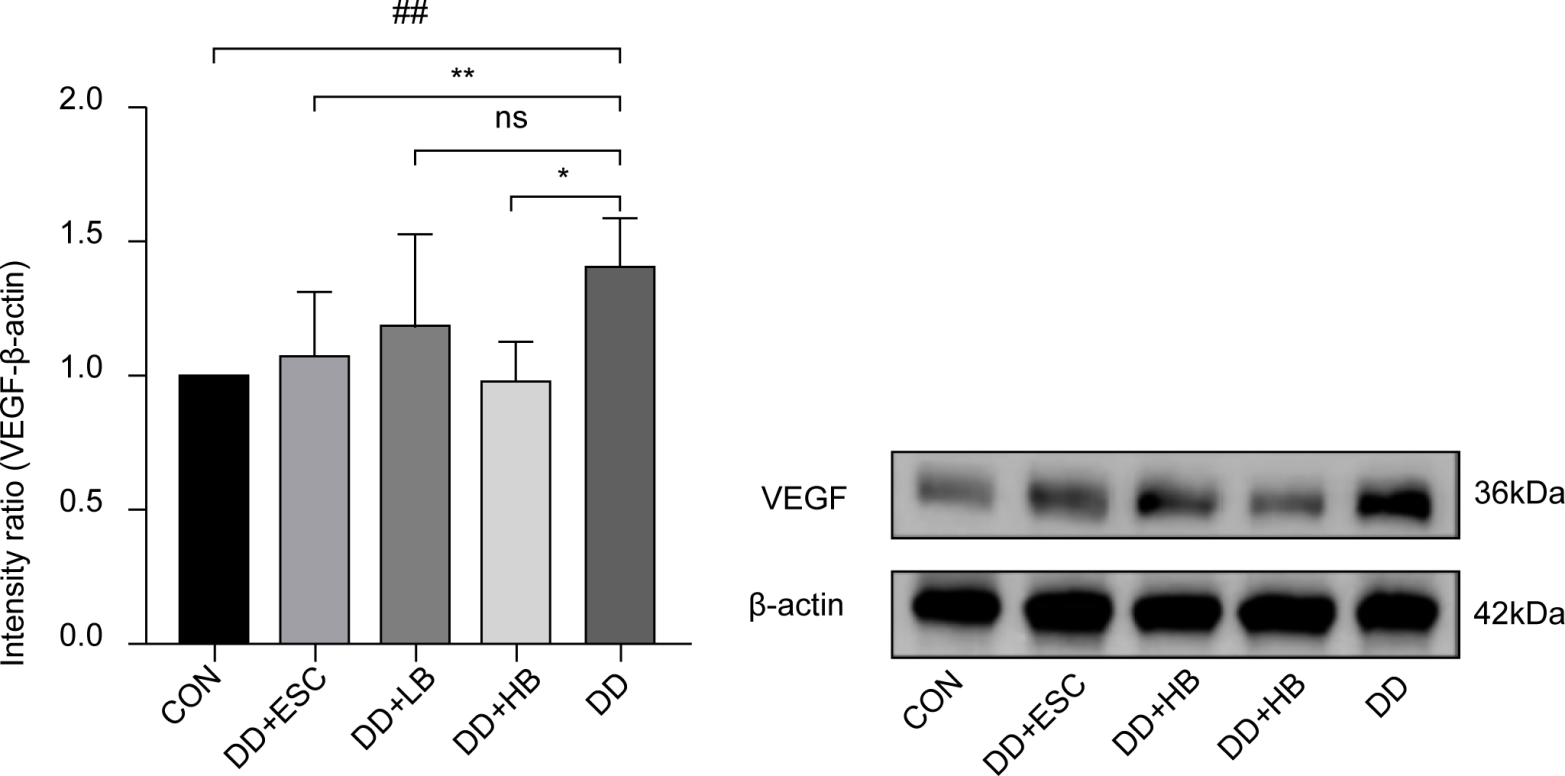
Changes in VEGF protein expression in the placenta of mice in each group. CON, control group; DD, depression model group; DD+HB, depression model + high-dose betaine group; DD+LB, depression model + low-dose betaine group; DD+ESC, depression model + escitalopram group. ##Compared with the control group, P < 0.01; *Compared with the depression model group, P < 0.05; **Compared with the depression model group, P < 0.01.

**Figure 7:**
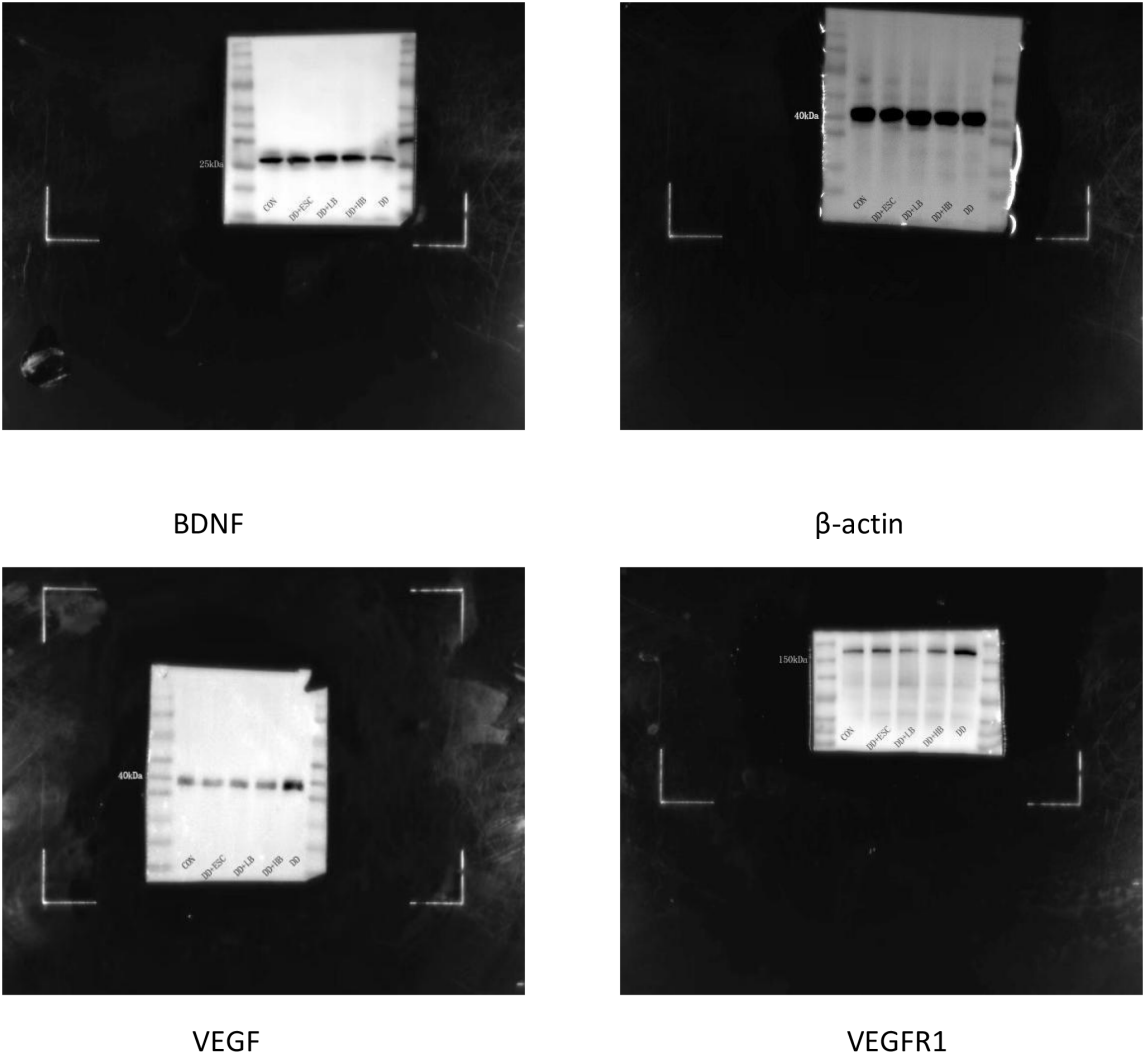
Western blot membranes for VEGF, VEGFR1, BDNF, HIF-1α, and β-actin.

Placental VEGF protein was upregulated in depressed dams (P < 0.01 vs. control). High-dose betaine attenuated this increase (P < 0.05), low-dose betaine had no effect (P > 0.05), and escitalopram restored VEGF to near-control levels (P < 0.01) (**Figures 6**, **7**).

#### 3.3.2 Placental VEGFR1 protein expression

**Figure 8:**
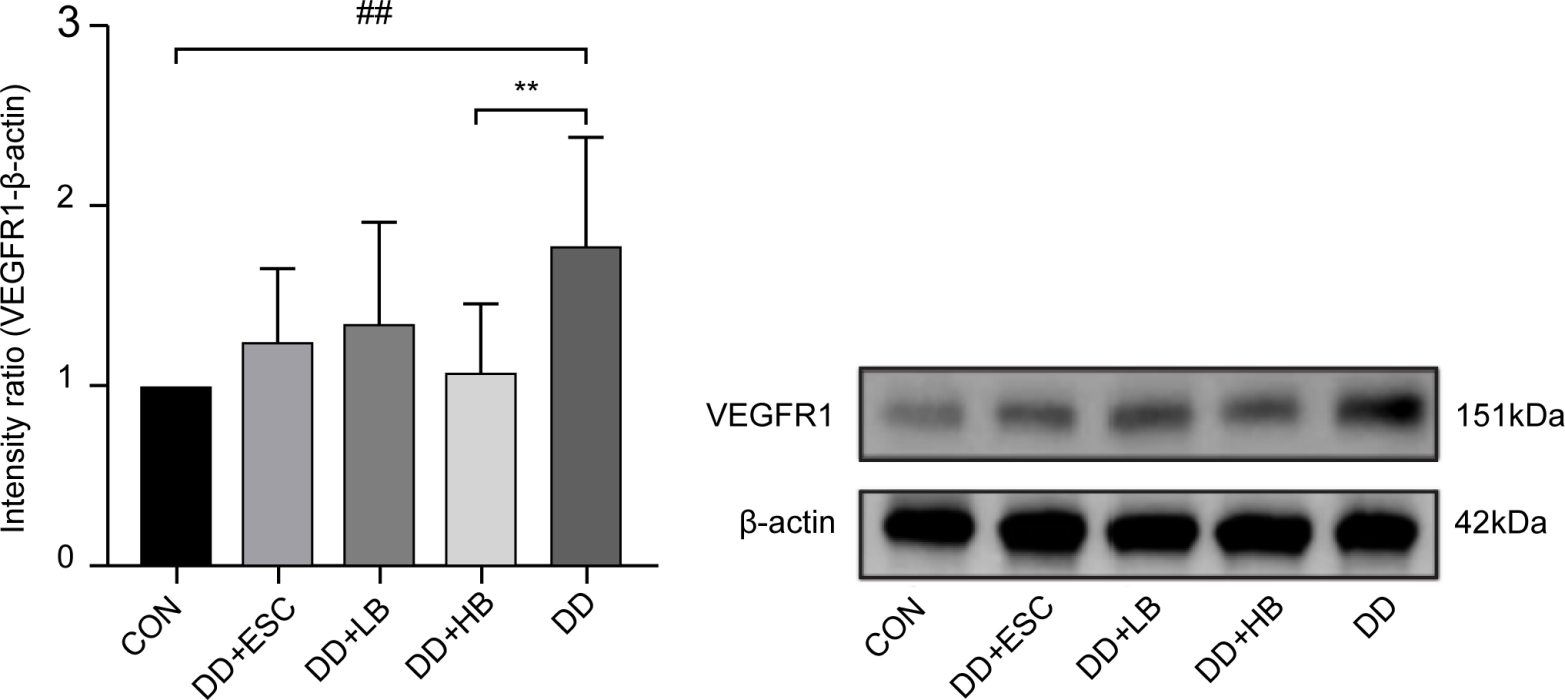
Changes in VEGFR1 protein expression in the placenta of mice in each group. CON, control group; DD, depression model group; DD+HB, depression model + high-dose betaine group; DD+LB, depression model + low-dose betaine group; DD+ESC, depression model + escitalopram group. ##Compared with the control group, P < 0.01; **Compared with the depression model group, P < 0.01.

Placental VEGFR1 protein was upregulated in stressed dams (P < 0.01 vs. control). High-dose betaine reversed this rise (P < 0.01), whereas low-dose betaine and escitalopram left VEGFR1 levels unchanged (P > 0.05) (**Figures 7**, **8**).

#### 3.3.3 Placental BDNF protein expression

**Figure 9:**
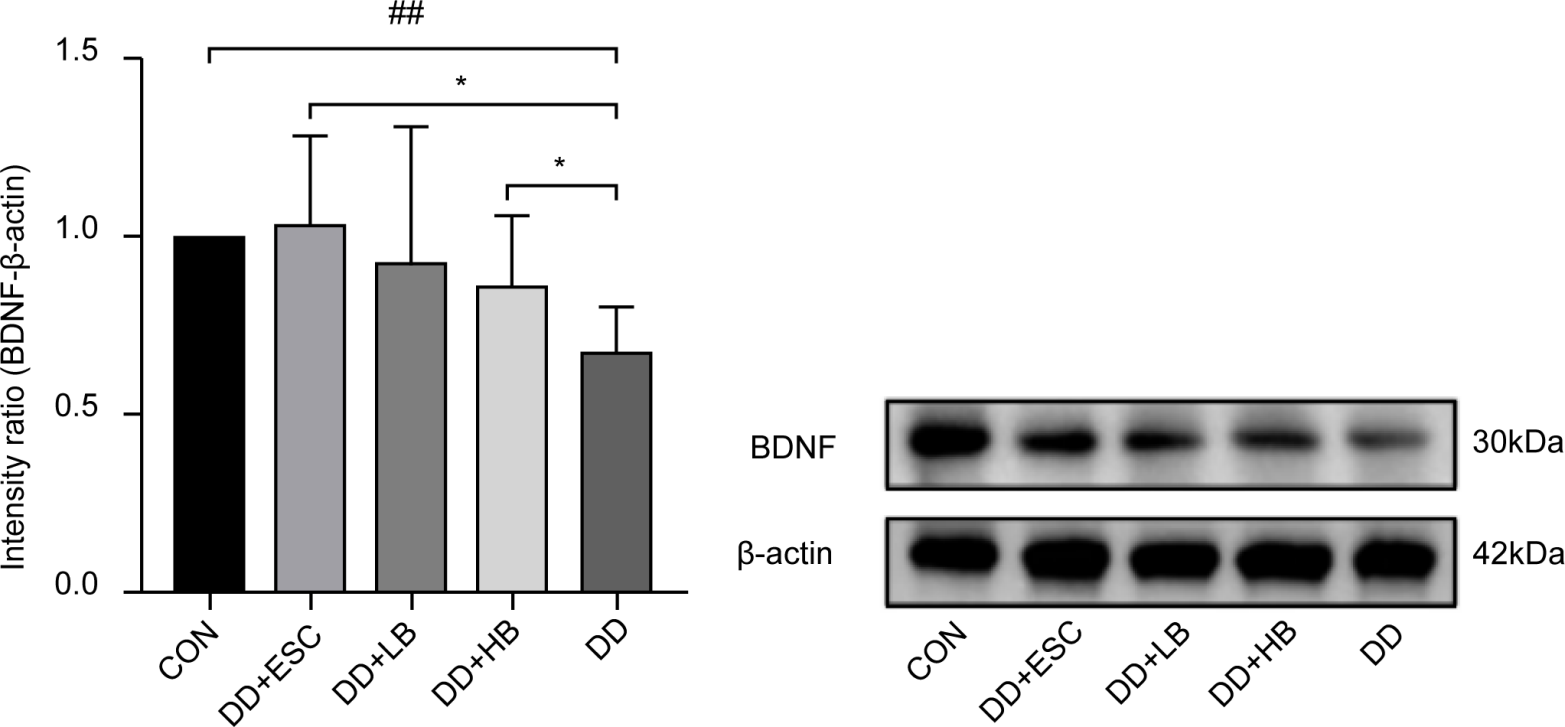
Changes in BDNF protein expression in the placenta of mice in each group. CON, control group; DD, depression model group; DD+HB, depression model + high-dose betaine group; DD+LB, depression model + low-dose betaine group; DD+ESC, depression model + escitalopram group. ##Compared with the control group, P < 0.01; *Compared with the depression model group, P < 0.05.

Placental BDNF protein was downregulated in stressed dams (P < 0.01 vs. control). High-dose betaine restored this loss (P < 0.05), whereas low-dose betaine left BDNF levels unchanged (P > 0.05) and escitalopram partially rescued the deficit (P < 0.05) (**Figures 7**, **9**).

### 3.4 mRNA expression levels of VEGF, BDNF, VEGFR1, and HIF-1α in the placenta of PND mice

#### 3.4.1 Placental VEGF mRNA expression

**Figure 10:**
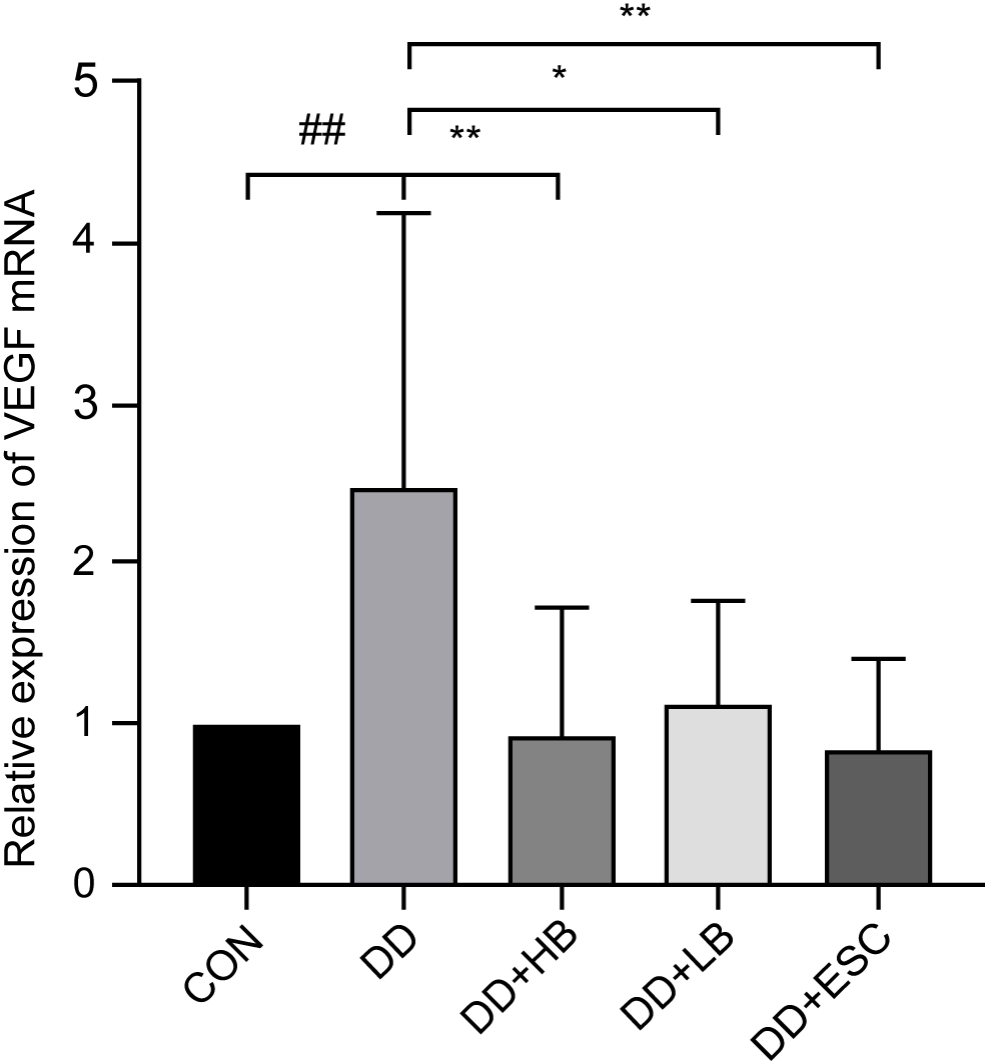
Changes in VEGF mRNA expression in the placenta of mice in each group. CON, control group; DD, depression model group; DD+HB, depression model + high-dose betaine group; DD+LB, depression model + low-dose betaine group; DD+ESC, depression model + escitalopram group. ##Compared with the control group, P < 0.01; *Compared with the depression model group, P < 0.05. ** Compared with the depression model group, P < 0.01.

VEGF transcript levels were significantly elevated in placentas from depressed dams compared to controls (P < 0.01). High-dose betaine attenuated this increase (P < 0.01), low-dose betaine resulted in a smaller reduction (P < 0.05), and escitalopram reduced VEGF mRNA to a similar extent as high-dose betaine (P < 0.01) (**Figure 10**).

#### 3.4.2 Placental VEGFR1 mRNA expression

**Figure 11:**
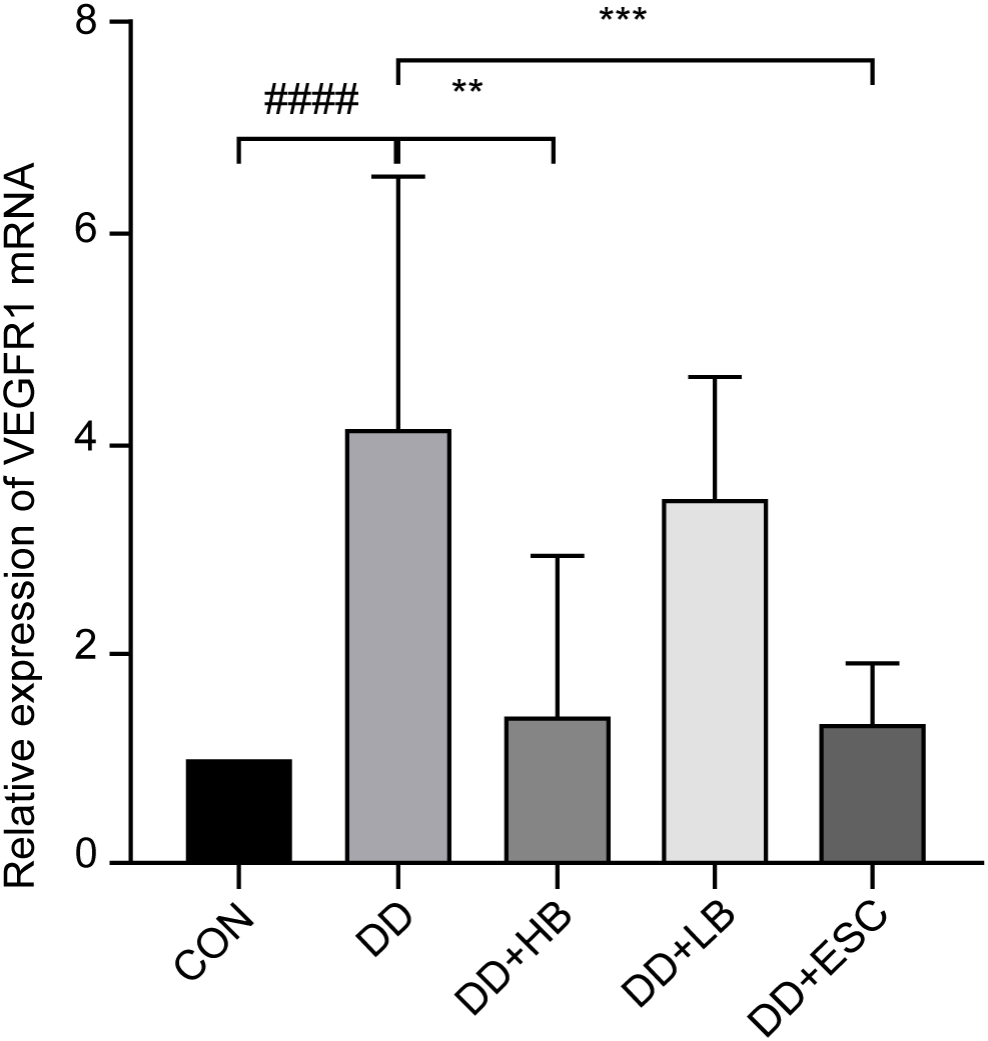
Changes in VEGFR1 mRNA expression in the placenta of mice in each group. CON, control group; DD, depression model group; DD+HB, depression model + high-dose betaine group; DD+LB, depression model + low-dose betaine group; DD+ESC, depression model + escitalopram group. **Compared with the depression model group, P < 0.01. ###Compared with the control group, P < 0.001; *Compared with the depression model group, P < 0.01; ***Compared with the depression model group, P < 0.001.

Placental VEGFR1 mRNA was higher in stressed dams than in controls (P < 0.001). High-dose betaine reduced transcript levels (P < 0.01), low-dose betaine left them unchanged (P > 0.05), and escitalopram produced a further reduction (P < 0.001) (**Figure 11**).

#### 3.4.3 Placental BDNF mRNA expression

**Figure 12:**
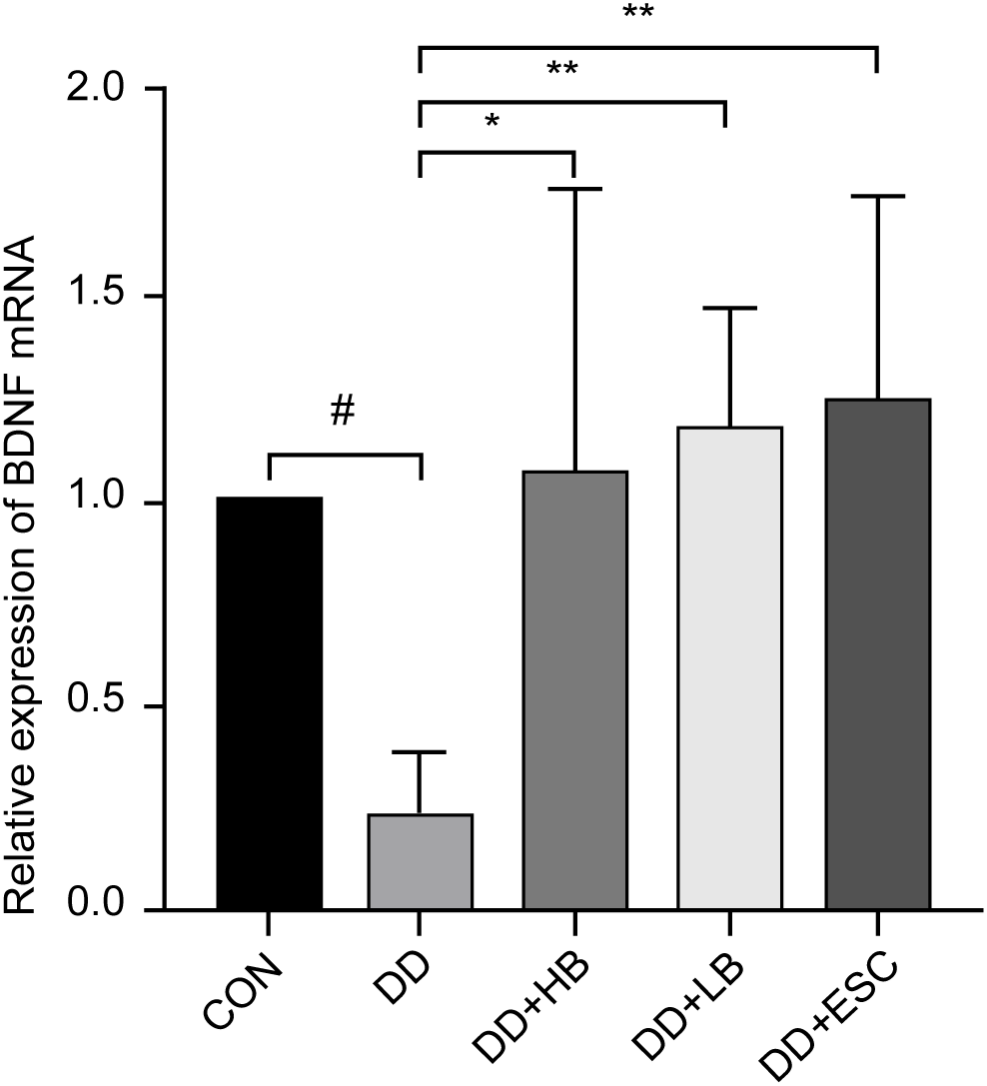
Changes in BDNF mRNA expression in the placenta of mice in each group. CON, control group; DD, depression model group; DD+HB, depression model + high-dose betaine group; DD+LB, depression model + low-dose betaine group; DD+ESC, depression model + escitalopram group. #Compared with the control group, P < 0.05; *Compared with the depression model group, P < 0.05; **Compared with the depression model group, P < 0.01.

Placental BDNF transcripts were significantly downregulated in depressed dams compared to controls (P < 0.05). This reduction was reversed by all interventions: high-dose betaine (P < 0.05), low-dose betaine, and escitalopram (both P < 0.01) (**Figure 12**).

#### 3.4.4 Placental HIF-1α mRNA expression

**Figure 13:**
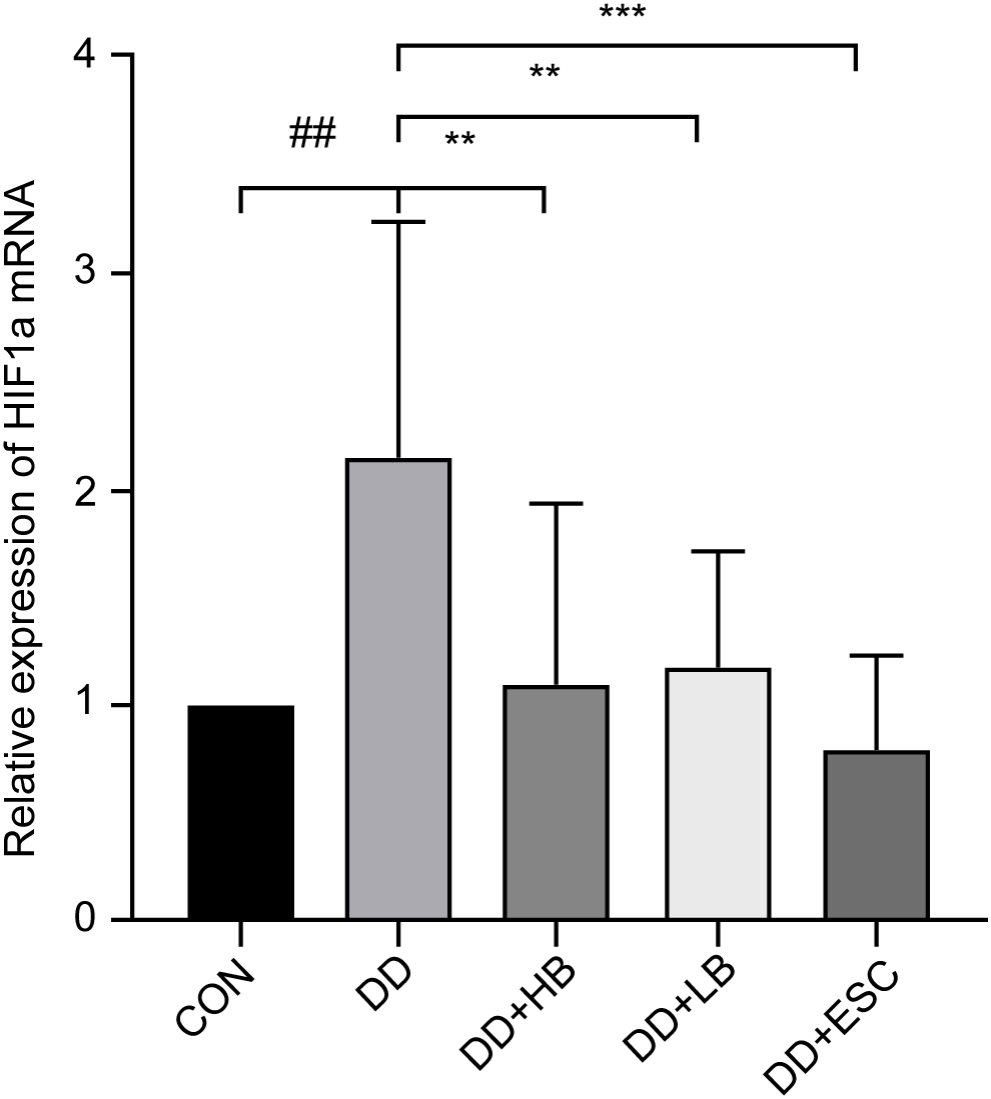
Changes in HIF1a mRNA expression in the placenta of mice in each group. CON, control group; DD, depression model group; DD+HB, depression model + high-dose betaine group; DD+LB, depression model + low-dose betaine group; DD+ESC, depression model + escitalopram group. ##Compared with the control group, P < 0.01; **Compared with the depression model group, P < 0.01; ***Compared with the depression model group, P < 0.001.

Compared with the control group, HIF-1α mRNA expression in the depression model cohort showed a marked elevation (P < 0.01). Relative to the depression model group, HIF-1α mRNA expression was markedly decreased in the depression model + high-dose betaine group (P < 0.01), significantly reduced in the depression model + low-dose betaine group (P < 0.05), and substantially lowered in the depression model + escitalopram group (P < 0.001) (**Figure 13**).

### 3.5 IHC findings in the placenta of perinatal depression mice

#### 3.5.1 Localization and expression of CD34 in the placenta

**Figure 14:**
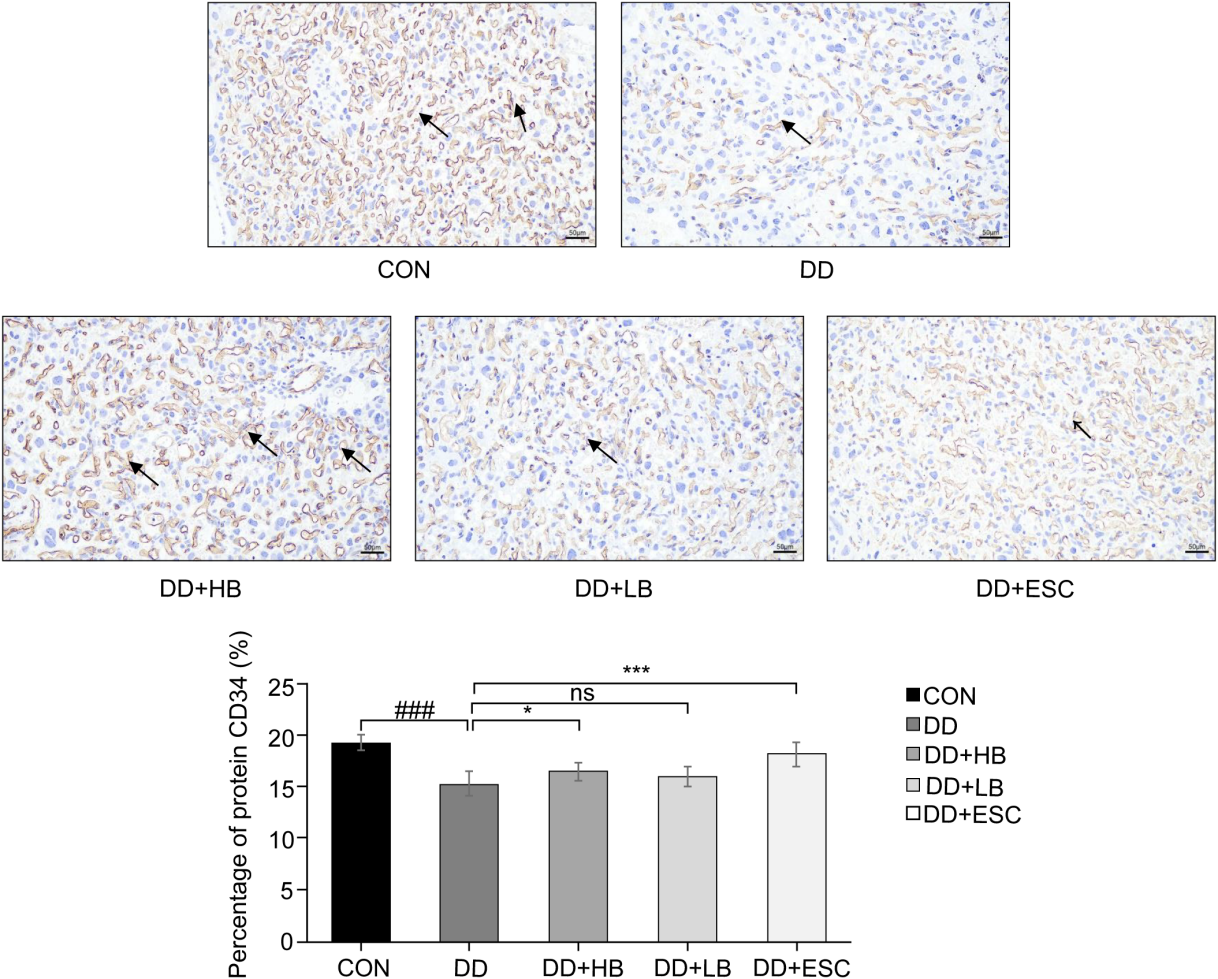
Comparison of CD34 expression in placental tissues of each group. Brown staining indicates CD34-positive endothelial cells (arrows point to representative microvessels). Original magnification ×200. CON, control group—CD34 expression in placental tissue (×200). DD, depression model group—CD34 expression in placental tissue (×200). DD+HB, depression model + high-dose betaine group—CD34 expression in placental tissue (×200). DD+LB, depression model + low-dose betaine group—CD34 expression in placental tissue (×200). DD+ESC, depression model + escitalopram group—CD34 expression in placental tissue (×200). ###Compared with the control group, P < 0.001; *Compared with the depression model group, P < 0.05; ***Compared with the depression model group, P < 0.001.

CD34 immunostaining revealed brown vascular signals against blue hematoxylin backgrounds in all cohorts, with reactivity confined to endothelial cells. CD34 expression, quantified as the percentage of immunopositive brown-stained area per high-power field using ImageJ, was lowest in depressed dams (P < 0.001 vs. control), rebounded after high-dose betaine (P < 0.05) and escitalopram (P < 0.001) treatment, but remained unaltered with low-dose betaine (P > 0.05) (**Figure 14**).

#### 3.5.2 Comparison of MVD among the five placental tissue groups

**Figure 15:**
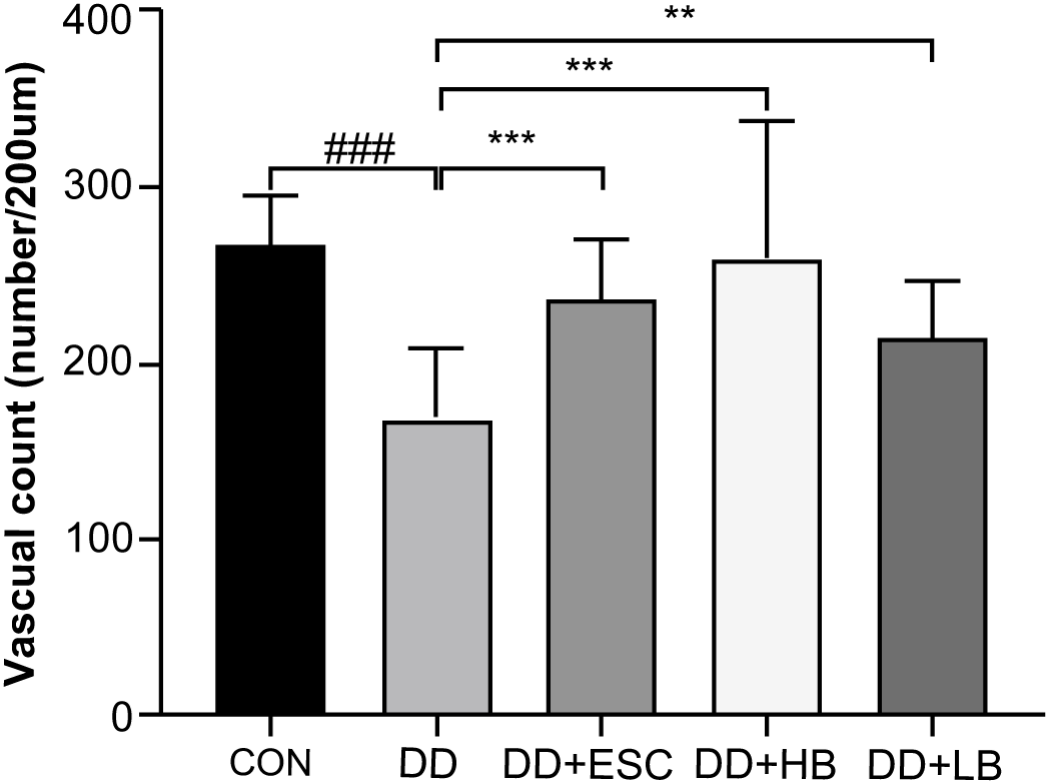
Comparison of microvascular density (MVD) in placental tissues of each group. CON, control group (cells/200 µm); DD, depression model group (cells/200 µm); DD+HB, depression model + high-dose betaine group (cells/200 µm); DD+LB, depression model + low-dose betaine group (cells/200 µm); DD+ESC, depression model + escitalopram group (cells/200 µm). ###Compared with the control group, P < 0.001; **Compared with the depression model group, P < 0.01; ***Compared with the depression model group, P < 0.001.

IHC with an anti-CD34 monoclonal antibody was used to label vascular endothelial cells and quantify MVD in placental tissues. MVD, determined by counting individual CD34-positive vessels with distinct lumens, showed a significant reduction in the model group compared with the control group (P < 0.001). Relative to the model group, MVD increased sharply in the high-dose betaine-treated model group (P < 0.001), increased modestly in the model + low-dose betaine group (P < 0.01), and was significantly elevated in the model + escitalopram group (P < 0.001) (**Figure 15**).

### 3.6 Comparison of histopathological changes in placental tissue among groups

**Figure 16:**
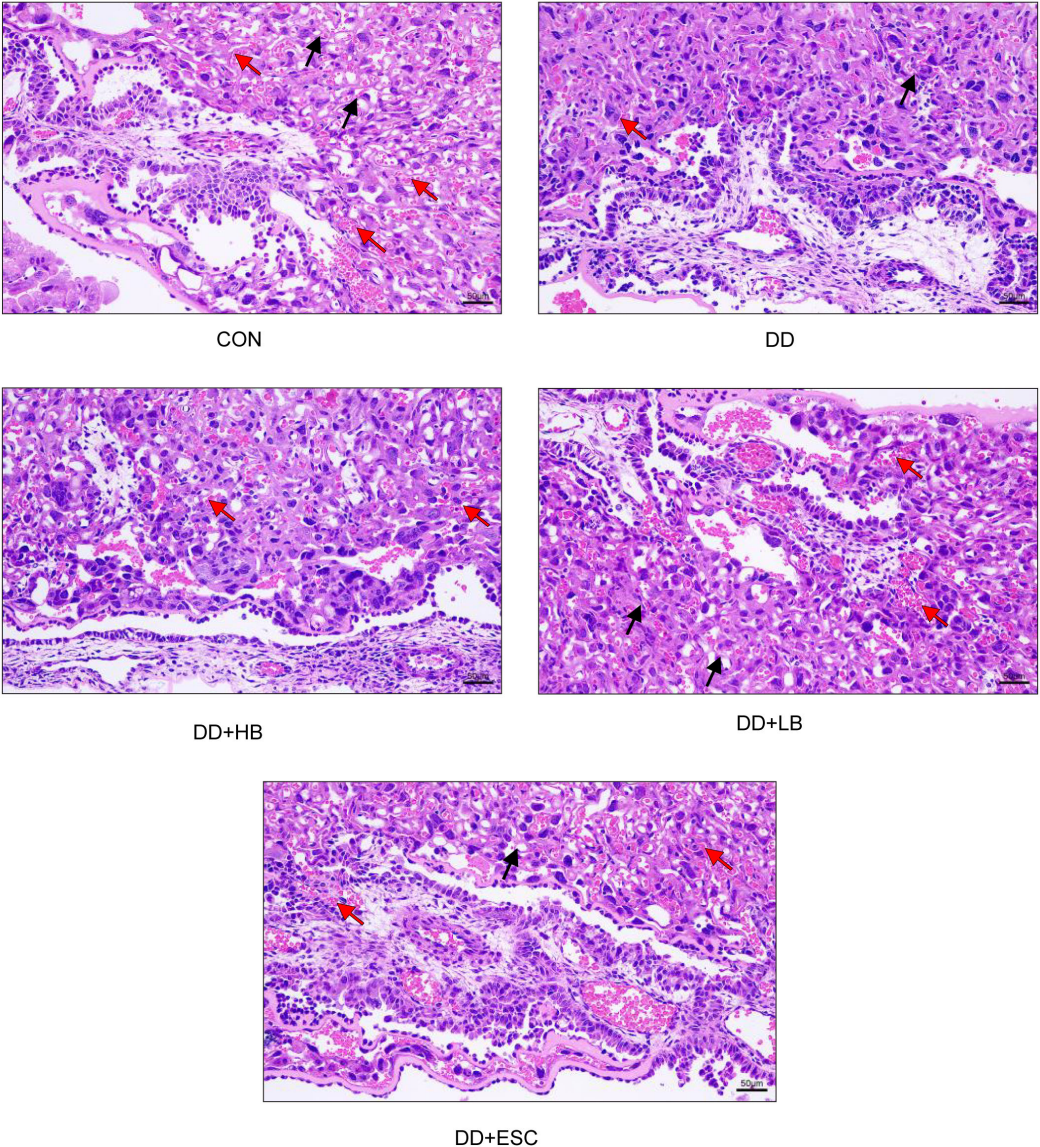
Comparison of pathological changes in the placental tissue of each group. CON, control group, showing regular vascular arrangement (black arrows) and abundant blood cells (red arrows). DD, depression model group, showing disorganized vascular distribution and reduced blood cells. DD+HB, depression model + high-dose betaine group, showing restored vascular architecture. DD+LB, depression model + low-dose betaine group, showing partial improvement. DD+ESC, depression model + escitalopram group, showing improved vascular arrangement. Black arrows indicate placental blood vessels (vessels with distinct lumens and endothelial lining); red arrows indicate blood cells (eosinophilic, anucleated red structures).

Hematoxylin-eosin staining revealed blue-stained nuclei and red-stained cytoplasm and erythrocytes. Compared with the control group, the model group showed a disordered distribution of placental blood vessels and a marked reduction in blood cells. In contrast, the model + high-dose betaine, model + low-dose betaine, and model + escitalopram groups all exhibited more regularly arranged vessels and an evident increase in blood cells relative to the model group (**Figure 16**).

## 4 Discussion

Our CUMS plus social isolation model is consistent with previously established perinatal depression models in rodents. For instance, Laviola and colleagues demonstrated that pre-gestational stress in rats induces depressive-like behaviors and alters maternal care, while studies by Franklin and colleagues showed that chronic stress during pregnancy in mice leads to HPA axis dysregulation and adverse offspring outcomes. Compared to these models, our approach combining 6 weeks of pre-mating CUMS with continued isolation through mid-gestation provides a robust and clinically relevant framework that captures both the chronicity and unpredictability of stress exposure characteristic of human perinatal depression.

As the etiology of PND remains poorly understood and human studies are limited by ethical and confounding constraints, we established a mouse model combining CUMS with individual housing. Unpredictable stressors evoke depressive-like states, whereas social isolation amplifies the stress response (3–8, 12–17). Six weeks of pre-mating exposure, continued until gestational day 14, produced robust behavioral deficits—anhedonia, hypoactivity, and behavioral despair—thereby providing a validated framework for mechanistic and interventional investigation.

Stressed dams exhibited reduced reactivity, weight loss, dull fur, reduced sucrose preference, decreased center exploration, and increased immobility in the forced-swim test (all P < 0.05), fulfilling standard criteria for a depressive phenotype in pregnancy. The depressive-like phenotype observed in our stressed dams—characterized by reduced sucrose preference, increased immobility in the forced swim test, and decreased exploratory behavior in the open field—is consistent with established CUMS-induced behaviors reported in the literature. Specifically, our findings align with those of Willner et al., who originally described sucrose preference deficits following chronic mild stress, and the more recent studies of Monteiro et al. in C57BL/6 mice, showing similar behavioral despair and anhedonia. The magnitude of behavioral changes in our study (approximately 30-40% reduction in sucrose preference, 2-fold increase in immobility time) is comparable to, or greater than, that reported in previous perinatal stress models, supporting the validity of our approach.

Neurotrophic factors, including BDNF, VEGF, and glial cell line-derived neurotrophic factor (GDNF), modulate mood and vascular plasticity (66). Although hippocampal and serum BDNF are consistently reduced in major depression (66), placental BDNF and its impact on fetal growth in PND remain unexplored. VEGF is elevated during acute depressive episodes (67) but falls below healthy levels after prolonged antidepressant treatment (68). Our finding of reduced placental BDNF protein and mRNA in PND dams contrasts with the elevated serum BDNF sometimes reported in acute depressive episodes, but it aligns with chronic stress paradigms showing downregulation of neurotrophic support. This reduction is particularly significant given the demonstration by Xiaofang et al. that decreased placental BDNF in gestational diabetes impairs labyrinthine angiogenesis and fetal growth. Our observation that high-dose betaine restored BDNF to control levels suggests that normalization of placental neurotrophic signaling may underlie its beneficial effects on fetal growth, extending the findings of Kim et al. who showed antidepressant-like effects of betaine in non-pregnant rodents.

HIF-1α, stabilized under hypoxic conditions, transcriptionally activates VEGF and VEGFR1 (69–71). VEGFR1 (Flt-1) lacks a functional kinase domain and acts as a decoy receptor that sequesters VEGF, thereby inhibiting VEGFR2-mediated angiogenesis (72, 73). In pre-eclampsia, concurrent upregulation of HIF-1α, VEGF, and VEGFR1 fails to enhance vascularization because soluble VEGFR1 blocks VEGF–VEGFR2 interaction, leading to systemic endothelial dysfunction and FGR (74–76). BDNF–TrkB signaling further promotes placental endothelial proliferation; however, reduced placental BDNF in mice with gestational diabetes mellitus decreases angiogenesis and distorts labyrinthine architecture, thereby compromising fetal growth (77). The concurrent elevation of VEGF, VEGFR1, and HIF-1α in PND placentas, despite reduced microvascular density, mirrors the pathological angiogenic profile observed in preeclampsia and IUGR pregnancies. This pattern is consistent with the “angiogenic imbalance” hypothesis described by Maynard et al., where excess soluble VEGFR1 (sFlt-1) antagonizes VEGF signaling despite high ligand levels. Our histological findings of disordered vascular architecture and reduced perfusion further support this interpretation. The ability of high-dose betaine to normalize these parameters—reducing HIF-1α, VEGF, and VEGFR1 while increasing CD34-positive vessels—suggests restoration of physiological angiogenic homeostasis. This dual action of betaine, previously demonstrated in pathological retinal neovascularization by Park et al., appears to extend to placental tissue, where it may suppress hypoxia-driven pathological signaling while promoting adaptive vascular repair.

Elevated placental HIF-1α mRNA suggests hypoxic conditions in the CUMS plus isolation model (78). The mechanism likely involves stress-induced uteroplacental vasoconstriction and reduced blood flow. Chronic stress activates the hypothalamic–pituitary–adrenal (HPA) axis and sympathetic nervous system, leading to catecholamine release and uterine artery constriction (79). Additionally, stress-induced oxidative stress and endothelial dysfunction may compromise placental vascular function (80). While we did not directly measure uteroplacental blood flow in this study, future investigations using Doppler ultrasound or microsphere perfusion techniques are warranted to confirm reduced placental perfusion as the primary driver of hypoxia.

In the present study, CUMS combined with isolation elevated placental HIF-1α, VEGF, and VEGFR1; however, CD34-positive vessel density and MVD were paradoxically decreased. Thus, VEGFR1-mediated inhibition outweighed VEGF-driven pro-angiogenic signaling, resulting in impaired vascularization, reduced placental efficiency, and FGR. These findings mirror clinical observations linking untreated PND to low birthweight and adverse neurodevelopmental outcomes (12–17, 56).

CD34 is selectively expressed on placental endothelial cells and serves as a quantitative marker of villous microvessels (81, 82). CD34^+^ cells secrete pro-angiogenic factors and replenish the endothelial pool; their depletion impairs vascularization. In a recurrent spontaneous abortion mouse model, diminished placental CD34 and reduced vessel density compromised fetal perfusion (83). In our study, PND placentas exhibited lower CD34 and decreased MVD, confirming that depression restricts placental vascularity. Both betaine and escitalopram restored CD34 expression and increased MVD, indicating that pharmacological intervention can reverse PND-induced vascular rarefaction.

Under hypoxic conditions, betaine suppresses pathological neovascularization by inhibiting ROS-mediated VEGF signaling (including reductions in VEGFR-2 and Akt/ERK phosphorylation) and attenuating VEGFA transcription (44). Under physiological conditions, it scavenges free radicals, elevates SOD, lowers MDA, upregulates Bcl-2, downregulates Bax, inhibits endothelial apoptosis, and promotes endothelial proliferation and migration (82). By enhancing nitric oxide synthesis, betaine improves vasodilation and placental perfusion, thereby supporting adaptive angiogenesis. Thus, betaine exerts context-dependent dual effects, being anti-angiogenic in pathological neovascularization yet pro-angiogenic during physiological repair.

Consistent with this paradigm, high-dose betaine reversed PND-induced elevations in HIF-1α, VEGF, and VEGFR1, increased CD34 and MVD, and augmented placental BDNF mRNA and protein. Consequently, placental efficiency and fetal weight were restored. The concentration-dependent efficacy observed (high dose > low dose) highlights the need for dose optimization in future translational studies.

The CUMS model incompletely recapitulates the heterogeneous pathology of human PND. Hence, placenta-specific HIF-1α manipulations and longitudinal neurobehavioral follow-up of offspring are required to validate molecular targets and trans-generational impact. Specifically, future studies should (i) develop placenta-specific HIF-1α inhibitors to dissect causal pathways, (ii) explore the synergistic effects of betaine with existing antidepressants, (iii) quantify fetal brain BDNF levels and assess long-term neurobehavioral outcomes, and (iv) clarify VEGF–BDNF interactions in placental development and pathology. We recognize that HIF-1α is primarily regulated post-translationally via protein stabilization under hypoxic conditions. While our qPCR data demonstrate increased HIF-1α transcription, future studies should include Western blot analysis to confirm protein-level accumulation and nuclear translocation as definitive evidence of placental hypoxia.

Additionally, a major limitation of the current study is the lack of direct assessment of maternal stress hormones. While the CUMS plus isolation model has been validated in numerous studies to induce HPA axis activation and elevate circulating corticosterone and ACTH levels (55), we did not measure these parameters in our experimental cohort. The absence of circulating stress hormone data limits our ability to confirm the severity of stress experienced by the dams and to correlate stress intensity with placental pathological changes. Future studies should incorporate serial measurements of plasma corticosterone, ACTH, and other stress-related biomarkers (such as pro-inflammatory cytokines and oxidative stress markers) to establish dose–response relationships between stress exposure, hormonal changes, and placental dysfunction.

## 5 Conclusions

A mouse model of PND was successfully established using CUMS combined with social isolation. Dams exhibited robust depressive- and anxiety-like behaviors, and their fetuses exhibited marked intrauterine growth restriction.

PND precipitated placental hypoxia, evidenced by HIF-1α accumulation and dysregulated VEGF/VEGFR1 signaling. This imbalance suppressed placental angiogenesis, reflected by reduced CD34 expression and MVD, decreased placental efficiency, and culminated in FGR.

High-dose betaine reversed these pathological alterations by attenuating HIF-1α over-activation, restoring VEGF/VEGFR1 equilibrium, and enhancing placental vascularization. Consequently, betaine improved placental efficiency and mitigated FGR, highlighting its potential as a safe, mechanism-based intervention for depression-related placental dysfunction.

## Data Availability

The datasets presented in this article are not readily available due to privacy restrictions outlined in the ethics approval. Requests to access the datasets should be directed to Jing Chen, 494205690@qq.com.
